# Applying Machine Learning to Carotid Sonographic Features for Recurrent Stroke in Patients With Acute Stroke

**DOI:** 10.3389/fcvm.2022.804410

**Published:** 2022-01-28

**Authors:** Shih-Yi Lin, Kin-Man Law, Yi-Chun Yeh, Kuo-Chen Wu, Jhih-Han Lai, Chih-Hsueh Lin, Wu-Huei Hsu, Cheng-Chieh Lin, Chia-Hung Kao

**Affiliations:** ^1^Graduate Institute of Biomedical Sciences, College of Medicine, China Medical University, Taichung, Taiwan; ^2^Division of Nephrology and Kidney Institute, China Medical University Hospital, Taichung, Taiwan; ^3^Center of Augmented Intelligence in Healthcare, China Medical University Hospital, Taichung, Taiwan; ^4^Department of Computer Science and Engineering, National Chung Hsing University, Taichung, Taiwan; ^5^Graduate Institute of Biomedical Electronics and Bioinformatics, National Taiwan University, Taipei, Taiwan; ^6^Department of Family Medicine, China Medical University Hospital, Taichung, Taiwan; ^7^Division of Pulmonary and Critical Care Medicine, China Medical University Hospital and China Medical University, Taichung, Taiwan; ^8^Department of Nuclear Medicine and Positron Emission Tomography Center, China Medical University Hospital, Taichung, Taiwan; ^9^Department of Bioinformatics and Medical Engineering, Asia University, Taichung, Taiwan

**Keywords:** machine learning, carotid sonographic features, recurrent stroke, acute stroke, CatBoost model

## Abstract

**Background:**

Although carotid sonographic features have been used as predictors of recurrent stroke, few large-scale studies have explored the use of machine learning analysis of carotid sonographic features for the prediction of recurrent stroke.

**Methods:**

We retrospectively collected electronic medical records of enrolled patients from the data warehouse of China Medical University Hospital, a tertiary medical center in central Taiwan, from January 2012 to November 2018. We included patients who underwent a documented carotid ultrasound within 30 days of experiencing an acute first stroke during the study period. We classified these participants into two groups: those with non-recurrent stroke (those who has not been diagnosed with acute stroke again during the study period) and those with recurrent stoke (those who has been diagnosed with acute stroke during the study period). A total of 1,235 carotid sonographic parameters were analyzed. Data on the patients' demographic characteristics and comorbidities were also collected. Python 3.7 was used as the programming language, and the scikit-learn toolkit was used to complete the derivation and verification of the machine learning methods.

**Results:**

In total, 2,411 patients were enrolled in this study, of whom 1,896 and 515 had non-recurrent and recurrent stroke, respectively. After extraction, 43 features of carotid sonography (36 carotid sonographic parameters and seven transcranial color Doppler sonographic parameter) were analyzed. For predicting recurrent stroke, CatBoost achieved the highest area under the curve (0.844, CIs 95% 0.824–0.868), followed by the Light Gradient Boosting Machine (0.832, CIs 95% 0.813–0.851), random forest (0.819, CIs 95% 0.802–0.846), support-vector machine (0.759, CIs 95% 0.739–0.781), logistic regression (0.781, CIs 95% 0.764–0.800), and decision tree (0.735, CIs 95% 0.717–0.755) models.

**Conclusion:**

When using the CatBoost model, the top three features for predicting recurrent stroke were determined to be the use of anticoagulation medications, the use of NSAID medications, and the resistive index of the left subclavian artery. The CatBoost model demonstrated efficiency and achieved optimal performance in the predictive classification of non-recurrent and recurrent stroke.

## Introduction

Stroke is the second most common cause of death and a leading cause of disability worldwide (1). It is a heterogeneous syndrome with two major types: ischemic, which accounts for ~60–85% of all cases, and hemorrhagic. The common pathogenesis of both types involves atherosclerosis and hypertension (2, 3). Stroke may lead to a wide range of complications including neurological disorders, infections, mobility dysfunction, thromboembolism, and emotional disorders (4). Among these complications, recurrent stroke is considered the most catastrophic: patients with recurrent stroke often become trapped in a vicious cycle and experience rapid degradation of their functions (5, 6).

Studies have focused on the exploration and identification of risk factors for recurrent stroke, including left atrial enlargement (7); blood biomarkers (8, 9); elevated von Willebrand factor levels (10); and clinical factors such as components of metabolic syndrome (11), a history of coronary heart disease (12), frequent rehabilitation (13), and plaque and perfusion being visible in magnetic resonance imaging (MRI) (14–17). In addition to risk factors, risk scores—including the total small vessel disease score (18), simple point scores (19), the CHA2DS2VASc Score, the Essen Stroke Risk Score, and the ABCD3 serial score (20–22)—have been proposed for evaluating patients at risk of recurrent stroke. In studies that have investigated the components of these risk scores (18–22), imaging components have been reported to be as important as clinical components. Regarding the ABCD3 serial score, Kiyohara et al. discovered that adding intracranial arterial stenosis could further improve the score's predictive value for recurrent stroke (22). Although computed tomography and MRI are sensitive and accurate neuroimaging tools, they are often expensive and dependent on practitioners' interpretation skills, and their utilization rates vary widely (23, 24). Carotid Doppler sonography provides a low-cost, low-risk, and highly portable alternative modality for evaluating the vessels of patients with acute stroke (25). Although individual components, such as significant stenosis (>60%) determined by a patient's internal carotid artery (ICA)/common carotid artery (CCA) peak systolic velocity (PSV) ratio, have been reported to be effective carotid Doppler sonographic indicators (26), few large-scale studies have explored the use of machine learning analysis of carotid sonographic features for predicting recurrent stroke. We conducted a retrospective cohort study involving the collection of clinical and Doppler parameters and the application of machine learning models to differentiate between recurrent and non-recurrent stroke. We employed the CatBoost and Light Gradient Boosting Machine (LGBM) machine learning algorithms, which are seldom employed in medical studies. We also compared the performance of the random forest, support vector machine (SVM), decision tree, Logistic Regression, CatBoost, and LGBM algorithms.

## Methods

### Data Collection and Study Design

The electronic medical records of the enrolled subjects were retrospectively collected from the data warehouse of China Medical University Hospital (CMUH), a tertiary medical center in central Taiwan, from January 2012 to November 2018. The records contained longitudinal electronic demographic information, laboratory data, *International Classification of Diseases* (*ICD*) coding, records of medical procedures, and medical imaging (including computed tomography, MRI, ultrasounds, and nuclear imaging) for all inpatients and outpatients of CMUH. Most treatments by CMUH, especially those for catastrophic illnesses, were covered by Taiwan's National Health Insurance, and the medical payments were thus under strict supervision by the National Health Insurance Administration. This study was approved by the Research Ethics Committee of CMUH (CMUH109-REC2-035).

### Participants and Definitions

We included patients who were diagnosed with acute first stroke and who received a documented carotid ultrasound within 30 days of the acute stroke during the study period. Initially, we enrolled 10,822 patients. Patients who lacked a documented report of their carotid sonography or who lacked complete DICOM SR data for the carotid sonography were excluded. Each patient's carotid and transcranial color-coded sonographic parameters were collected and examined. Carotid sonography was performed on a GE Vivid 7 system (GE Healthcare, Milwaukee, WI, USA) with a 3–10 MHZ linear array transducer linear 9L probe.

**Figure 1:**
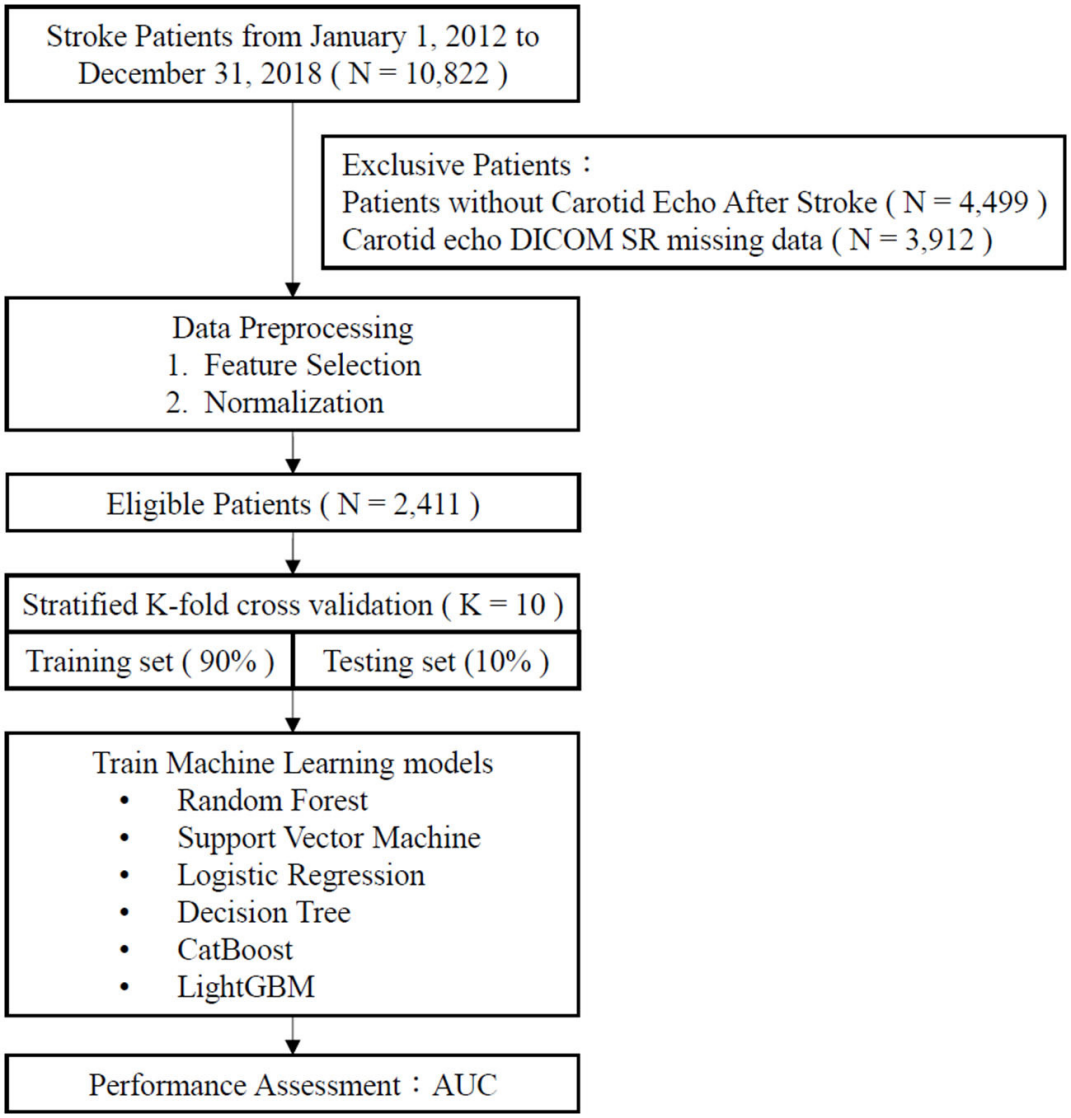
Flow chart.

The enrolled patients were classified into two groups: those who experienced non-recurrent stroke (stroke once; those who has not been diagnosed with acute stroke again) and those who experienced recurrent stroke (those who has been diagnosed with another acute stroke during study period; Figure 1).

In this study, acute stroke was defined according to the National Health Insurance Administration's definition of catastrophic illness *ICD-9* and *ICD-10* codes for acute stroke, including occlusion and stenosis, hemorrhagic strokes, transient ischemic attacks (TIAs) and related syndromes, stroke syndromes, and other cerebral vascular diseases (Supplementary Table 1). Comorbidities considered in this study included hypertension, diabetes mellitus, hyperlipidemia, end-stage renal disease, atrial fibrillation, heart failure, liver cirrhosis, and cancer, which were also defined based on *ICD* coding in the CMUH data warehouse.

The primary outcome of our study was recurrent stroke, and we established a model for predicting a patient's risk of recurrent stroke after their first episode of acute stroke.

### Data Preprocessing

Data preprocessing was required to ensure the performance of our model. We applied feature extraction and data normalization when preprocessing the collected clinical, demographic, and sonographic variables.

#### Feature Extraction

To identify significant features, three types of feature extraction were utilized in our study. First, we used Pearson correlation coefficients to determine the strength of the linear relationships between the carotid sonographic parameters and the clinical variables. The carotid sonographic parameters with Pearson correlation coefficients < 0.1 were not considered. Second, we used least absolute shrinkage and selection operator (lasso) regression, a shrinkage and variable selection method for regression models, to eliminate less representative features and select more representative features (27). Last, we used the statistical significance to determine significant features, which the carotid sonographic parameters with *p*-value < 0.05 were considered. Only the features selected using the Pearson correlation, lasso regression methods and statistical significance method were included in our training dataset.

#### Data Normalization

Because the units of the sonographic variables varied, data scaling was required for normalization. We applied standard deviation (SD) normalization, which is a method commonly used when a dataset contains a few non-extreme outliers. We calculated the mean and SD values of the training data and scaled the values to ensure that the mean of all the values was 0 and the SD was 1. The formula used to calculate the *z*-score was


$$\begin{array}{l} Z=\frac{x-\mu }{\sigma } \end{array}$$

where *x* is the original datum, μ is the mean, and σ is the SD.

#### Data Balancing

The ratio of patients with non-recurrent stroke to those with recurrent stroke was ~3:1. Therefore, the number of fault samples and the number of positive training samples were imbalanced, and the algorithm tended to ignore small classes and concentrate on the accurate classification of the large classes, resulting in a weaker model with limited predictive ability. To overcome the imbalanced nature of the data, we applied class weight balancing and balanced bagging methods in our training models. When class weight balancing methods are applied, if the sample size of a category is high, then it is assigned a low weight, and vice versa (28). Balanced bagging, which involves bootstrapping or applying sampling techniques to the original data *n* times with replacements to create training sets, also improves a model's classification accuracy and reduces data imbalance (29).

### Machine Learning Models

Six machine learning models were used in this study: random forest, SVM, Logistic Regression, decision tree, CatBoost, and LGBM.

**Random forest**, an ensemble learning technique, involves the aggregation of a large number of decision trees (30). Each individual tree in the random forest provides a class prediction based on a given number *mtry* of randomly selected features (31). Random forests produce less variance compared with single decision trees and produce predictions more accurate than those of any of the individual trees.

**SVMs** are linear supervised classifiers capable of performing binary and multiclass classification on a dataset (32). In an SVM, each data point is an *n*-dimensional vector. According to the margin maximization principle, the SVM chooses the most appropriate hyperplane to maximize the distance from the hyperplane to the nearest data point on each side (33).

**Logistic Regression**, a linear regression model, converts the log-odds of input variables to a predicted probability of outcome.

**Decision trees**, non-parametric supervised learning tools, are treelike structures consisting of a root node, condition or leaf nodes, and associated branches (34). The end of each branch that does not split anymore represents a potential outcome. The probability model with the maximum likelihood of attaining a desirable outcome among the decision trees was considered the most effective prediction model.

**CatBoost** is a gradient boosting framework that employs oblivious decision trees as base predictors; it is an open-source software library developed by Yandex (35). For each level of each decision tree, decision rules containing feature indices and threshold values are collected, which eventually form a collection of disjoint subsets of feature vectors. The collections of feature vectors function as a prediction model. **CatBoost** reduces overfitting and improves the quality of a model (36).

**LGBM** is another gradient boosting algorithm and an implementation of ensemble learning. LGBM uses a leaf-wise algorithm to grow trees vertically; a leaf that most reduces the loss is chosen to split (37). The main function of LGBM is to create large gradients, which contribute more to information gain (38).

### Statistical Analysis

Baseline sociodemographic and clinical characteristics are displayed as the mean ± SD. Categorical variables are expressed as absolute and percent frequencies.

The Python 3.7 software package and scikit-learn toolkit were employed, and the defaults were applied for the training of the random forest, SVM, LogisticRegression, decision tree, CatBoost, and LGBM algorithms. We used the Gaussian radial basis function as the kernel function in our SVM model, and the regularization parameter (*C*) was 1.0. For the random forest and decision tree algorithms, 10 decision trees were used. For the Logistic Regression algorithm, we added a penalty term (known as the L2 norm or L2 penalty) to the loss function. For the CatBoost algorithm, 1,000 decision trees and six hidden layers were used. For the LGBM, 100 decision trees and 31 hidden layers were used.

We used the following evaluation metrics of sensitivity, specificity, accuracy, and area under the receiver operating characteristic curve (AUC) to evaluate the performance of the machine learning algorithms in this study:


$$\begin{array}{l} \text{Sensitivity}=\frac{TP}{\left(TP+FN\right)}\\ \text{Specificity}=\frac{TN}{\left(TN+FP\right)},\\ \text{Accuracy}=\frac{\left(TP+TN\right)}{\left(TP+FP+TN+FN\right)}, \end{array}$$

where *TP* denotes true positives; *FP*, false positives; *TN*, true negatives; and *FN*, false negatives. Precision was denoted by *TP*/(*TP* + *FP)*, and recall was denoted by *TP*/(*TP* + *FN)*.

We used stratified *k*-fold cross-validation to estimate the accuracy of the models. The data were first stratified and then split into *k* portions. In each *k* iteration, one portion was used as the test set, and the remaining *k* – 1 portions were used as training sets. Then, the model was fit to the training sets, and the performance of the model on the test set was evaluated. This procedure was repeated until each of the *k* subsets had served as the validation set. The average of the *k* performance measurements on the *k* validation sets was the cross-validated performance (39). In this study, we used stratified 10-fold cross-validation to estimate the accuracy, as generally recommended (40). The Shap algorithm was used to measure the contribution of features to predicting “non-recurrence” and “recurrence.” Shapley Additive explanation proposed by Lundberg and Lee is a method of explaining predictions based on the optimal Shapley value of game theory (41).

## Results

### Patient Population and Demographics

A total of 2,411 patients were enrolled in this study. Of these, 1,896 were classified into the non-recurrent stroke cohort, and 515 were classified into the recurrent stroke cohort (Figure 1). The mean ages of the non-recurrent stroke and recurrent stroke cohorts were 66.18 ± 12.67 years (range: 24–98 years) and 67.63 ± 13.14 years (range: 27–96 years), respectively. Regarding gender, 61.66 and 62.14% of the patients in the non-recurrent stroke and recurrent stroke cohorts, respectively, were men. Strokes involving occlusion and stenosis were the most common and accounted for 75.84 and 80.19% of patients' strokes in the non-recurrent stroke and recurrent stroke cohorts, respectively. TIAs and related conditions were the second most common stroke type, accounting for 8.81 and 6.80% of the patients' strokes in the non-recurrent stroke and recurrent stroke cohorts, respectively. The prevalence of each comorbidity was higher among patients in the recurrent stroke cohort than among patients with non-recurrent stroke: of the patients with recurrent stroke, 72.04% had hypertension, 45.24% had diabetes mellitus, 27.96% had hyperlipidemia, 4.08% had end-stage renal disease, 8.54% had atrial fibrillation, 12.43% had heart failure, 8.35% had cancer, and 36.50% had a body mass index (BMI) > 25. Furthermore, 57.12 and 74.76% of the patients in the non-recurrent stroke and recurrent stroke cohorts, respectively, took dihydropyridine derivatives; 91.67 and 97.09% of patients in the non-recurrent stroke and recurrent stroke cohorts, respectively took antiplatelet medications; and 62.29 and 73.98% of the patients in the non-recurrent stroke and recurrent stroke cohorts, respectively, had taken HMG-COA inhibitors (Table 1).

**Table 1:** Clinical characteristics in 2,411 study patients.

	**Non-recurrent stroke (%)**	**Recurrent stroke (%)**	***p*-value**
**Study patients**	*N* = 1,896	*N* = 515	
Men	1,169 (61.66%)	320 (62.14%)	0.843
Age	66.18 ± 12.67 (24–98)	67.63 ± 13.14 (27–96)	<0.05
**Stroke type**			
Occlusion and stenosis	1,438 (75.84%)	413 (80.19%)	<0.05
Hemorrhage	144 (7.59%)	30 (5.83%)	0.169
TIA and related syndrome	167 (8.81%)	35 (6.80%)	0.144
Stroke syndrome	51 (2.69%)	8 (1.55%)	0.139
Others cerebral vascular disease	96 (5.06%)	29 (5.63%)	0.606
**Comorbidity**			
Hypertension	1,227 (64.72%)	371 (72.04%)	<0.05
Diabetes mellitus	682 (35.97%)	233 (45.24%)	<0.001
Hyperlipidemia	503 (26.53%)	144 (27.96%)	0.516
End stage renal disease	10 (0.53%)	21 (4.08%)	<0.0001
Atrial fibrillation	81 (4.27%)	44 (8.54%)	<0.001
Heart failure	123 (6.49%)	64 (12.43%)	<0.0001
Liver cirrhosis	36 (1.90%)	7 (1.36%)	0.412
Cancer	113 (5.96%)	43 (8.35%)	0.051
BMI > 25	675 (35.60%)	188 (36.50%)	0.862
Medicine after first stroke			
Angiotensin II receptor blockers (ARBs)	753 (39.72%)	287 (55.73%)	<0.0001
Dihydropyridine derivatives	1,083 (57.12%)	385 (74.76%)	<0.0001
Anti-coagulant	665 (35.07%)	353 (68.54%)	<0.0001
Anti-platelet	1,738 (91.67%)	500 (97.09%)	<0.0001
HMG-COA inhibitors	1,181 (62.29%)	381 (73.98%)	<0.0001
NSAID	761 (40.14%)	312 (60.58%)	<0.0001

*Values are expressed as the mean ± SD.

*HTN, hypertention; DM, diabetes mellitus; ESRD, end stage renal disease*.

### Selected Features

The algorithms identified 36 carotid sonographic parameters and seven transcranial color-coded sonographic parameter as features. Supplementary Table 2 summarizes the results of the feature selection process of sonographic parameters; bold type denotes the selected features. In addition, 18 clinical variables, namely age, gender, stroke type, hypertension, diabetes mellitus, hyperlipidemia, end-stage renal disease, atrial fibrillation, heart failure, liver cirrhosis, cancer, and types of medications, were identified as features. In total, this study involved the analysis of 65 features.

### Performance of Models in Predicting Non-recurrent and Recurrent Stroke

**Figure 2:**
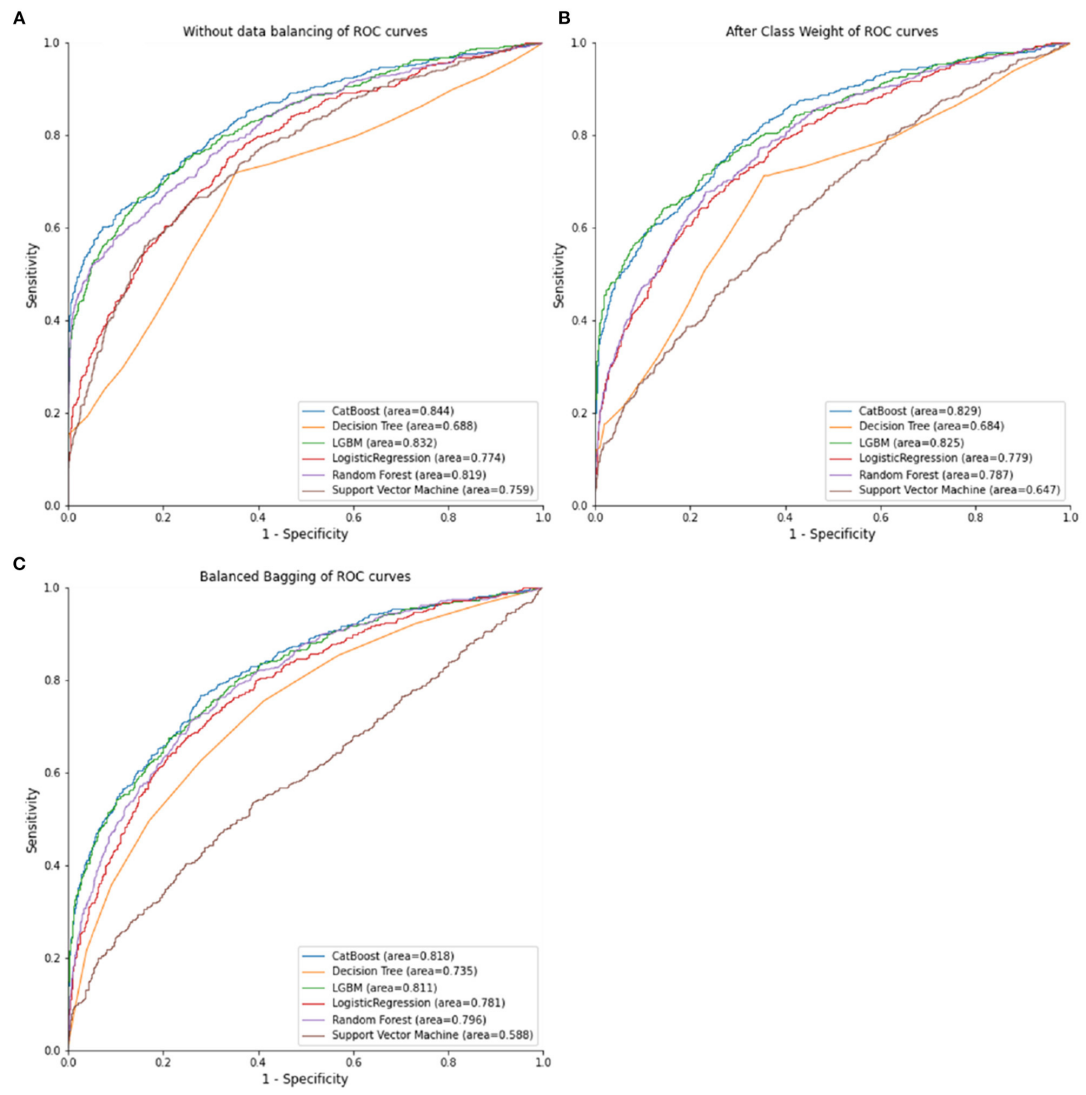
ROC curves of combinations of different data balancing methods and machine learning algorithms. **(A)** No data balancing. **(B)** After class weight. **(C)** balanced bagging.

Table 2 listed the predictive performance of the random forest, SVM, LogisticRegression, decision tree, CatBoost, and LGBM models. The best AUC achieved was 0.844 (0.824–0.868) by the CatBoost model with no balancing method, exceeding the AUC of 0.818 (0.797–0.843) achieved by the CatBoost model with class weight balancing and the AUC of 0.829 (0.814–0.849) achieved by the CatBoost model with balanced bagging. The random forest model (AUC = 0.819, 0.802–0.846), CatBoost model (AUC = 0.844, 0.824–0.868), and LGBM model (AUC = 0.832, 0.813–0.851) resulted in higher AUCs without balancing methods than when class weight balancing or balanced bagging were employed. The Logistic Regression model (AUC = 0.781, 0.764–0.800) and decision tree model (AUC = 0.735, 0.717–0.755) resulted in higher AUCs with balanced bagging methods than when either of non-balancing or class weight balancing methods were employed. Figure 2 illustrates the receiver operating characteristic curve of the random forest, SVM, Logistic Regression, decision tree, CatBoost, and LGBM models in combination with the various data balancing methods.

**Table 2:** Comparison of the predictive performance for six models (cross-validated data).

**Model**	**Method**	**Sensitivity**	**Specificity**	**Accuracy**	**AUC**
		**(CIs 95%)**	**(CIs 95%)**	**(CIs 95%)**	**(CIs 95%)**
RF	-	0.070 (0.049–0.091)	1.000 (1.000–1.000)	0.801 (0.797–0.805)	0.819 (0.802–0.846)
SVM	-	0.216 (0.184–0.247)	0.973 (0.968–0.978)	0.811 (0.805–0.818)	0.759 (0.739–0.781)
LR	-	0.305 (0.256–0.353)	0.958 (0.948–0.969)	0.819 (0.809–0.829)	0.774 (0.759–0.793)
DT	-	0.155 (0.116–0.194)	0.997 (0.994–1.000)	0.817 (0.811–0.824)	0.688 (0.686–0.733)
CatBoost	-	0.441 (0.394–0.488)	0.994 (0.990–0.998)	0.876 (0.867–0.885)	0.844 (0.824–0.868)
LGBM	-	0.421 (0.381–0.461)	0.982 (0.977–0.987)	0.862 (0.855–0.870)	0.832 (0.813–0.851)
RF	Class weight	0.678 (0.632–0.722)	0.762 (0.742–0.782)	0.744 (0.726–0.762)	0.787 (0.766–0.818)
SVM	Class weight	0.060 (0.038–0.082)	0.996 (0.993–0.999)	0.796 (0.791–0.801)	0.647 (0.617–0.683)
LR	Class weight	0.678 (0.636–0.720)	0.735 (0.707–0.763)	0.723 (0.703–0.743)	0.779 (0.762–0.798)
DT	Class weight	0.717 (0.664–0.769)	0.624 (0.606–0.641)	0.644 (0.627–0.661)	0.684 (0.674–0.726)
CatBoost	Class weight	0.522 (0.484–0.560)	0.928 (0.912–0.943)	0.841 (0.832–0.851)	0.829 (0.814–0.849)
LGBM	Class weight	0.493 (0.467–0.519)	0.954 (0.943–0.965)	0.856 (0.845–0.866)	0.825 (0.808–0.843)
RF	Balanced bagging	0.604 (0.558–0.649)	0.814 (0.790–0.838)	0.769 (0.751–0.788)	0.796 (0.770–0.823)
SVM	Balanced bagging	0.474 (0.432–0.516)	0.675 (0.636–0.714)	0.632 (0.602–0.662)	0.588 (0.563–0.620)
LR	Balanced bagging	0.687 (0.640–0.734)	0.736 (0.706–0.765)	0.725 (0.707–0.744)	0.781 (0.764–0.800)
DT	Balanced bagging	0.497 (0.463–0.531)	0.829 (0.815–0.842)	0.758 (0.752–0.764)	0.735 (0.717–0.755)
CatBoost	Balanced bagging	0.606 (0.555–0.656)	0.845 (0.823–0.867)	0.794 (0.780–0.808)	0.818 (0.797–0.843)
LGBM	Balanced bagging	0.592 (0.559–0.625)	0.851 (0.835–0.866)	0.796 (0.786–0.805)	0.811 (0.793–0.833)

Based on the models' calculated accuracy, the CatBoost model without a balancing method exhibited the optimal performance in predicting the patients' risk of recurrent stroke (accuracy = 0.844), followed by the LGBM without balancing methods (accuracy = 0.832), the LGBM model without balancing methods (accuracy = 0.839), and the CatBoost model with class weight balancing (Accuracy = 0.829). Regarding specificity, the random forest with no balancing methods achieved the highest specificity (1.000).

The performance of each training model, as judged by sensitivity, was inadequate without the application of balancing methods. After data balancing using the class weight or balanced bagging methods, the sensitivity of random forest, Logistic Regression, decision tree, CatBoost, and LGBM models increased; the decision tree model with class weight balancing achieved a sensitivity of 0.617.

**Figure 3:**
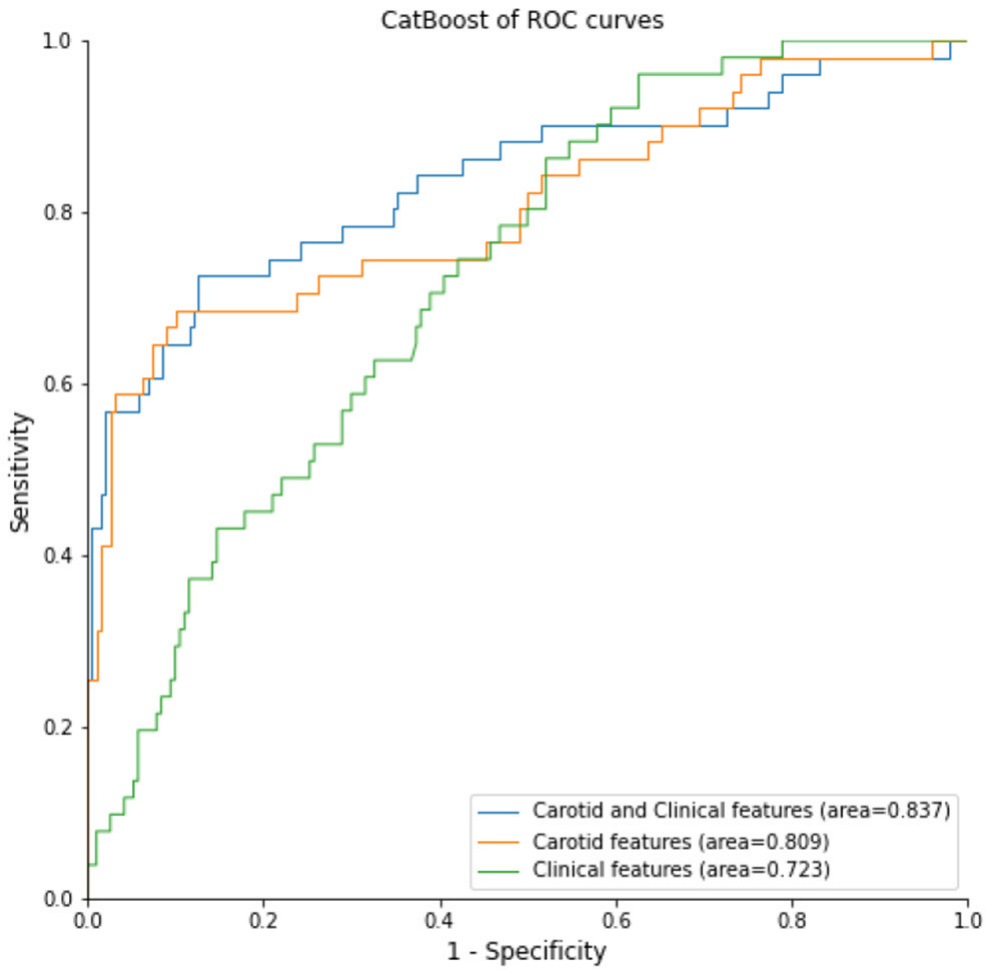
CatBoost model for different settings: clinical data, sonographic data, and combination of clinical and sonographic data for recurrent stroke prediction.

Since CatBoost model performed best among these models, we also compare the carotid sonographic features, clinical characteristics and combination between non-recurrent stroke and recurrent-stroke in CatBoostt model. Figure 3 showed AUC of combination was 0.837, AUC of carotid sonographic features was 0.809, and AUC of clinical parameters was 0.723.

### Top 10 Significant Features Correlated With Recurrent Stroke in the CatBoost Model

**Figure 4:**
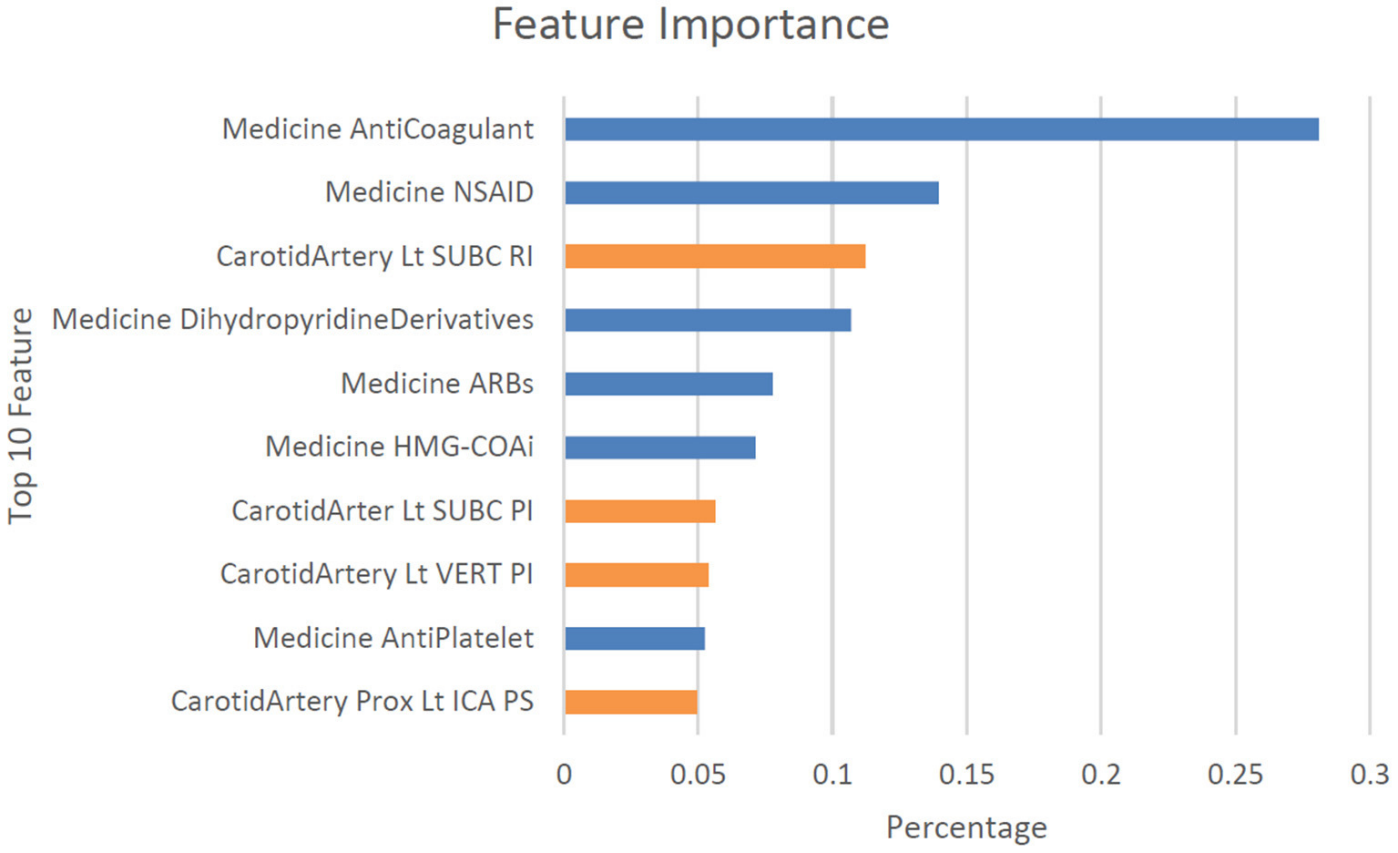
Top 10 features observed in the CatBoost model without balancing method for classifying stroke once and recurrent stroke. Dist, distal; Lt, left, Rt, right; CCA, common carotid artery; ICA, internal carotid artery; ECA, external carotid artery; SUBC, subclavian artery; VERT, vertebral artery; BA, basilar artery; Prox, proximal; TCD, transcranial Doppler; PI, pulse index; RI, resistive index; TAMEAN, intensity-weighted mean frequency; EDV, end-diastolic velocity; PSV, peak systolic velocity.

In this study, the CatBoost model with no balancing methods exhibited the best and the most stable performance, with an AUC of 0.844 (0.824–0.868), an accuracy of 0.876 (0.867–0.885), a sensitivity of 0.441 (0.394–0.488, no balancing method), and a specificity of 0.994 (0.990–0.998). We further analyzed the details of the CatBoost algorithm. The confusion matrix of the CatBoost model indicated that the numbers of patients with true positive and true negative results were 227 and 1,885, respectively, in our cross validated data set (Table 3). We also explored significant features identified by the CatBoost model for optimally predicting recurrent stroke. The top 10 most significant features were the use of anticoagulation medications, the use of NSAID medications, the resistive index (RI) of the left subclavian artery, the use of dihydropyridine derivatives medications, the use of ARBs medications, the use of HMG-COAi medications, the PI of the left subclavian artery, the PI of the left vertebral artery, the use of anti-platelet medications, and the PSV (peak systolic velocity) of the left proximal internal carotid artery (Figure 4).

**Table 3:** Confusion matrix for the CatBoost without balancing method prediction.

		**Predicted**	
		**No**	**Yes**
True	No	1,885	11
	Yes	288	227

## Discussion

In this study, we adopted machine learning algorithms to analyze potential clinical and sonographic risk factors for recurrent stroke among patients with acute stroke. We first evaluated the patients' carotid sonographic parameters using a large-scale CatBoost model and identified key features associated with an increased risk of recurrent stroke, including the use of anticoagulation medications, the use of NSAID medications, the resistive index (RI) of the left subclavian artery, the use of dihydropyridine derivatives medications, the use of ARBs medications, the use of HMG-COAi medications, the PI of the left subclavian artery, the PI of the left vertebral artery, the use of antiplatelet medications, and the PSV (peak systolic velocity) of the left proximal internal carotid artery. The CatBoost model demonstrated efficiency and achieved optimal performance in predicting non-recurrent and recurrent stroke on the basis of carotid Doppler sonographic parameters.

The significant correlation between the use of anticoagulation medications and recurrent stroke is reasonable because patients who have already experienced a stroke commonly use anticoagulation medications, especially those who had stroke of embolic events (42). Besides, our findings might also imply that cardiac or cryptogenic embolism would play a role in recurrent stroke (43, 44). Among the top 10 features correlated with recurrent stroke, four were carotid ultrasonographic parameters that require further investigation. Patients' vessel diameter; plaque; PSV; PI; RI; and end-diastolic velocity (EDV) of the left and right external carotid artery, internal carotid artery, subclavian artery, basilar artery, CCA, and vertebral artery are components commonly examined in standard carotid Doppler sonographic exams (45). However, most previous research has focused on the effects on stroke risk exerted by specific carotid sonographic features such as occlusion of the middle cerebral artery (MCA) (46); high-intensity signals of symptomatic arteries (47), carotid arteries, M1 segments of the MCA (48), and P2 segments of posterior cerebral arteries (PCAs) (48); the presence of carotid plaque (48, 49); ICA/CCA PSV ratios (50); decreased poststenotic PSV (51); and poststenotic arterial narrowing (51). To the best of our knowledge, this is the first study to involve the large-scale investigation of all carotid sonographic parameters. The correlation powers of the PI and RI of certain carotid arteries with recurrent stroke were greater than indices of carotid arteries stenosis, including PSV and percentage of stenosis, in our machine learning models. The degree of a patient's stenosis could be determined by their intima thickening and residual diameter/total diameter in grayscale ultrasound and PSV, ICA/CCA PSV, and ICA EDV in color Doppler ultrasound (52–54). Each patient's PI was calculated using the formula PI = (PSV – EDV)/MV, and the RI was calculated using RI = PSV – EDV/PSV (55). Previous studies have demonstrated that stroke risk is positively correlated with degree of stenosis in patients with symptomatic carotid stenosis (56). However, in the present study, we observed that the PI and RI of individual subclavian, vertebral, and internal carotid arteries were more positively correlated with recurrent stroke than stenosis was. These study results provide valuable clinical information because each carotid sonography was performed within 30 days of the respective patient's acute stroke (46). Our findings are consistent with those of Barnett et al. that ~20 and 45% of strokes in the symptomatic and asymptomatic carotid arteries with 70–99% stenosis, respectively, are unrelated to carotid stenosis (57). Because the complex machine learning algorithms employed in this study are black boxes, the variables explored should be interpreted as powerful indicators for differential diagnosis but not as casual factors (58). Furthermore, this study employed a cohort design; whether the PI and RI of individual subclavian, vertebral, and internal carotid arteries can be used to predict recurrent stroke must be investigated in future studies.

Regarding the machine learning algorithms, we believe we are the first to adopt the CatBoost model in the risk assessment of patients with acute stroke, and our study demonstrated that the CatBoost model exhibited high performance in predicting recurrent stroke. CatBoost is a powerful machine learning algorithm suitable for datasets with many categorical variables (59). CatBoost is commonly utilized in the fields of business (60), financial assessments (61), Medicare fraud detection (62), environmental science (63, 64), and public science (36). According to our review of the literature, in the field of medicine, the random forest model has retained a competitive edge and is often superior in the prediction and classification of medical conditions compared with traditional logistic regression methods and machine learning methods such as neural networks, SVMs, and decision trees (65–68). In our study, the CatBoost model outperformed the random forest model in classifying non-recurrent and recurrent stroke. Although its performance in other hospital settings has not been explored, our results indicate that CatBoost may be considered when selecting machine leaning models to apply for treating acute stoke or elsewhere in the medical field.

This study has several limitations. First, it is a single-center-based retrospective study, and external validation would be required to determine machine learning models' suitability for the risk assessment of other patients with acute stroke. Second, this was a retrospective cohort study and carotid ultrasonographic features were collected in the first time of stroke. Thus, the top 10 features identified in the study should be interpreted as predicative or classification factors rather than as casual factors. Further large-scale prospective cohort studies are necessary to investigate the predictive value of these features. Third, due to incomplete carotid Doppler sonography and missing values, only ~22% of 10,822 patients with acute stroke were enrolled in the study. Possible enrollment bias and baseline bias may have influenced the results. Fourth, information regarding the infarct areas of the patients was unavailable in the database employed in the study. Therefore, although we have reported four significant sonographic parameters related to brain vessels, whether the individual vessels overlapped with infarct areas or same infarct territory of high-risk artery were not analyzed in this study. Finally, in this study, further analysis about using TOAST classification for cerebral ischemic cases or dual antiplatelet agents among the recurrent event group have not been performed.

In conclusion, this study revealed that the CatBoost model is efficient and achieved optimal performance in predicting non-recurrent and recurrent stroke. The flow parameters of the carotid ultrasound, PI and RI, are more useful in differentiating between non-recurrent and recurrent stroke compared with other carotid ultrasonographic parameters.

## Data Availability Statement

The raw data supporting the conclusions of this article will be made available by the authors, without undue reservation.

## Ethics Statement

The studies involving human participants were reviewed and approved by the Research Ethics Committee of CMUH (CMUH109-REC2-035). Written informed consent for participation was not required for this study in accordance with the national legislation and the institutional requirements.

## Author Contributions

S-YL and C-HK: conception/design. C-HK: provision of study materials. All authors have contributed significantly, agreement with the content of the manuscript, collection and/or assembly of data, data analysis, interpretation, manuscript writing, and final approval of manuscript.

## Funding

This study was supported in part by China Medical University Hospital (DMR-110-089, DMR-111-090, and DMR-111-091); Ministry of Science and Technology (MOST 110-2321-B-039-003). The funders had no role in the study design, data collection and analysis, the decision to publish, or preparation of the manuscript. No additional external funding was received for this study.

## Conflict of Interest

The authors declare that the research was conducted in the absence of any commercial or financial relationships that could be construed as a potential conflict of interest.

## Publisher's Note

All claims expressed in this article are solely those of the authors and do not necessarily represent those of their affiliated organizations, or those of the publisher, the editors and the reviewers. Any product that may be evaluated in this article, or claim that may be made by its manufacturer, is not guaranteed or endorsed by the publisher.
