# Supplementary material for: Applying Machine Learning to Carotid Sonographic Features for Recurrent Stroke in Patients With Acute Stroke
Source: Front Cardiovasc Med. 2022 Jan 28;9:804410. doi: 10.3389/fcvm.2022.804410 (PMC8833232; doi:10.3389/fcvm.2022.804410)
Supplement: Supplementary file 1 [file Table_1.docx]

| Supplementary Table 1. DiagCode of StrokeType. | | | | | | |  |
| --- | --- | --- | --- | --- | --- | --- | --- |
|  | |  |  | |  |  |  |
| StrokeType | |  | Diagnostic Code | | | |  |
|  |  |  |  |  |  |  |  |
|  |  |  |  | |  |  |  |
|  |  |  | ICD9 | |  | ICD10 |  |
| Hemorrhage | |  | 430~432 | |  | I60~I62 |  |
|  | |  |  | |  |  |  |
| Occlusion and stenosis | |  | 433、434 | |  | I63、I65、I66 |  |
|  | |  |  | |  |  |  |
| TIA and related syndrome | |  | 435.2、435.9、437.7 | |  | G45.1~G45.2、G45.4、G45.9、I67.84 |  |
| Stroke syndrome | |  | 435.0~435.1、435.3、435.8 | |  | G45.0、G45.8、G46.0~G46.2 |  |
|  | |  |  | |  |  |  |
| Sequelae of cerebrovascular disease | |  | 438 | |  | I69 |  |
|  | |  |  | |  |  |  |
| Others cerebral vascular disease | |  | 436、437 (except 437.7) | |  | G46.3~G46.8、I67 (except I67.3*****、I67.83*****、I67.84)、I68 |  |
|  |  |  |  |  |  |  |  |
| ***exclusive ICD 10 : G45.3、I67.3、I67.83** | | | | | | | |
