# Supplementary material for: Applying Machine Learning to Carotid Sonographic Features for Recurrent Stroke in Patients With Acute Stroke
Source: Front Cardiovasc Med. 2022 Jan 28;9:804410. doi: 10.3389/fcvm.2022.804410 (PMC8833232; doi:10.3389/fcvm.2022.804410)
Supplement: Supplementary file 2 [file Table_2.docx]

| **Supplementary Table 2.** Carotid ultrasound Parameters in 2,411 Study Patients | | | |
| --- | --- | --- | --- |
|  |  |  |  |
|  | Non-recurrent stroke * | Recurrent stroke* | *p* value |
|  |  |  |  |
| **Carotid Artery** |  |  |  |
|  |  |  |  |
|  |  |  |  |
|  |  |  |  |
| Dist_Lt_CCA PI^&^ | 1.71 ± 0.36 | 1.83 ± 0.53 | < 0.0001 |
| Dist_Lt_CCA PS^&^ (cm / sec) | 61.04 ± 15.52 | 60.04 ± 17.42 | 0.18 |
| Dist_Lt_CCA RI^&^ | 0.78 ± 0.06 | 0.79 ± 0.07 | < 0.0001 |
| Dist_Lt_CCA TAMEAN^&^ (cm / sec) | 18.65 ± 5.23 | 17.8 ± 5.73 | < 0.01 |
| Dist_Lt_ICA EDV^&^ (cm / sec) | 25.39 ± 7.47 | 24.57 ± 9.09 | < 0.05 |
| Dist_Lt_ICA PI^&^ | 1.17 ± 0.25 | 1.3 ± 0.53 | < 0.0001 |
| Dist_Lt_ICA PSV (cm / sec) | 75.61 ± 17.72 | 75.84 ± 21.41 | 0.82 |
| Dist_Lt_ICA RI^&^ | 0.66 ± 0.07 | 0.67 ± 0.08 | < 0.01 |
| Dist_Rt_CCA PI^&^ | 1.75 ± 0.37 | 1.86 ± 0.5 | < 0.0001 |
| Dist_Rt_CCA PSV (cm / sec) | 54.38 ± 14.47 | 53.34 ± 15.97 | 0.16 |
| Dist_Rt_CCA RI^&^ | 0.79 ± 0.06 | 0.8 ± 0.07 | < 0.0001 |
| Dist_Rt_CCA TAMEAN^&^ (cm / sec) | 17.08 ± 4.95 | 16.39 ± 5.09 | < 0.01 |
| Dist_Rt_ICA EDV^&^ (cm / sec) | 24.52 ± 7.67 | 23.52 ± 8.4 | < 0.05 |
| Dist_Rt_ICA PI^&^ | 1.19 ± 0.25 | 1.33 ± 0.58 | < 0.0001 |
| Dist_Rt_ICA PS (cm / sec) | 74.95 ± 18.26 | 74.69 ± 22.23 | 0.73 |
| Dist_Rt_ICA RI^&^ | 0.67 ± 0.07 | 0.68 ± 0.09 | < 0.01 |
| Lt_ECA PI^&^ | 2.83 ± 0.79 | 3.02 ± 1.02 | < 0.0001 |
| Lt_ECA RI^&^ | 0.9 ± 0.06 | 0.91 ± 0.07 | < 0.001 |
| Lt_ECA TAMEAN^&^ (cm / sec) | 16.41 ± 4.84 | 17.25 ± 8.54 | < 0.01 |
| Lt_SUBC PI^&^ | 9.07 ± 3.2 | 10.05 ± 5.54 | < 0.0001 |
| Lt_SUBC RI^&^ | 1.01 ± 0.02 | 1 ± 0.05 | < 0.01 |
| Lt_SUBC TAMEAN^&^ (cm / sec) | 10.44 ± 4.7 | 11.15 ± 8.45 | < 0.05 |
| Lt_VERT PI^&^ | 1.54 ± 0.4 | 1.73 ± 0.85 | < 0.0001 |
| Lt_VERT RI^&^ | 0.75 ± 0.08 | 0.76 ± 0.1 | < 0.05 |
| Rt_ECA PI^&^ | 2.86 ± 0.78 | 3.09 ± 1.05 | < 0.0001 |
| Rt_ECA RI^&^ | 0.9 ± 0.06 | 0.91 ± 0.06 | < 0.01 |
| Rt_ECA TAMEAN (cm / sec) | 17.18 ± 5.26 | 17.31 ± 6.46 | 0.78 |
| Rt_SUBC PI^&^ | 10.31 ± 3.57 | 11.62 ± 7.7 | < 0.0001 |
| Rt_SUBC RI | 1.01 ± 0.02 | 1.01 ± 0.04 | 0.06 |
| Rt_SUBC TAMEAN^&^ (cm / sec) | 9.21 ± 4.01 | 9.26 ± 5.12 | 0.71 |
| Rt_VERT PI^&^ | 1.61 ± 0.44 | 1.77 ± 0.83 | < 0.0001 |
| Rt_VERT RI^&^ | 0.76 ± 0.08 | 0.77 ± 0.1 | 0.83 |
| Prox_Lt_ICA EDV^&^ (cm / sec) | 14.6 ± 4.93 | 15.15 ± 8.84 | 0.07 |
| Prox_Lt_ICA PI^&^ | 1.38 ± 0.34 | 1.45 ± 0.48 | < 0.001 |
| Prox_Lt_ICA PSV^&^ (cm / sec) | 50.75 ± 14.08 | 55.38 ± 29.43 | < 0.0001 |
| Prox_Lt_ICA RI^&^ | 0.71 ± 0.08 | 0.72 ± 0.09 | < 0.05 |
| Prox_Lt_ICA TAMEAN^&^ (cm / sec) | 16.72 ± 4.92 | 17.38 ± 7.55 | < 0.05 |
| Prox_Rt_ICA EDV ^&^ (cm / sec) | 14.32 ± 4.74 | 14.39 ± 6.47 | 0.8 |
| Prox_Rt_ICA PI^&^ | 1.37 ± 0.31 | 1.46 ± 0.53 | < 0.0001 |
| Prox_Rt_ICA PSV^&^ (cm / sec) | 50 ± 13.88 | 52.26 ± 20.87 | < 0.01 |
| Prox_Rt_ICA RI^&^ | 0.71 ± 0.07 | 0.72 ± 0.1 | < 0.01 |
| Prox_Rt_ICA TAMEAN (cm / sec) | 17.49 ± 5.17 | 17.59 ± 6.99 | 0.85 |
|  |  |  |  |
| TCD |  |  |  |
|  |  |  |  |
| Lt_VERT PI^&^ | 1.07 ± 0.23 | 1.13 ± 0.29 | < 0.0001 |
| Lt_VERT RI^&^ | 0.63 ± 0.08 | 0.64 ± 0.09 | < 0.01 |
| BA PI^&^ | 1.06 ± 0.23 | 1.09 ± 0.26 | < 0.01 |
| BA PSV^&^ (cm / sec) | 72.98 ± 26.28 | 78.46 ± 41.22 | < 0.001 |
| BA RI^&^ | 0.63 ± 0.08 | 0.64 ± 0.09 | < 0.05 |
| Rt_VERT PI^&^ | 1.12 ± 0.25 | 1.17 ± 0.34 | < 0.0001 |
| Rt_VERT RI^&^ | 0.64 ± 0.09 | 0.65 ± 0.1 | < 0.01 |
|  |  |  |  |

*Values are expressed as the mean ± SD. ^&^Parameters finally selected as training dataset in this table.

Dist, distal; Lt, left, Rt, right; CCA, common carotid artery; ICA, internal carotid artery; ECA, external carotid artery; SUBC, subclavian artery; VERT, vertebral artery; BA, basilar artery; Prox, proximal; TCD, transcranial Doppler; PI, pulse index; RI, resistive index; TAMEAN, Time-averaged mean velocity; EDV, end-diastolic velocity; PSV, peak systolic velocity
